# Ablation of PC1/3 in POMC-Expressing Tissues but Not in Immune Cells Induces Sepsis Hypersensitivity

**DOI:** 10.1210/jendso/bvae171

**Published:** 2024-10-03

**Authors:** Jana Moeller, Daniel T Meier

**Affiliations:** Clinic of Endocrinology, Diabetes and Metabolism, University Hospital Basel, 4031 Basel, Switzerland; Department of Biomedicine, University of Basel, 4031 Basel, Switzerland; Clinic of Endocrinology, Diabetes and Metabolism, University Hospital Basel, 4031 Basel, Switzerland; Department of Biomedicine, University of Basel, 4031 Basel, Switzerland

**Keywords:** prohormone convertase 1/3, sepsis, inflammation, hypothalamus-pituitary-adrenal axis, steroids

## Abstract

Prohormone convertase 1/3 (PC1/3) is an endopeptidase required for the processing of neuropeptide and endocrine peptide precursors; it is expressed in neuroendocrine tissues as well as in immune cells. In response to endotoxemia, global PC1/3 knockout mice mount a cytokine storm and die rapidly. Further, immune cells isolated from these mice have a pro-inflammatory signature, suggesting that PC1/3 activates an unknown anti-inflammatory peptide precursor in immune cells. Here, we tested this hypothesis using tissue-specific PC1/3 ablation models. Knocking out PC1/3 in the myeloid or the hematopoietic compartment did not induce any phenotype. In contrast, proopiomelanocortin (POMC)-specific PC1/3 knockout mice phenocopied global PC1/3 knockout mice, including an enlarged spleen size and a hyperinflammatory sepsis phenotype in response to mild endotoxemia. This phenotype was prevented by steroid therapy and mimicked by blocking corticoid receptors in wild-type mice. Thus, our data suggest that sepsis hypersensitivity in PC1/3 deficiency is uncoupled from immune cell intrinsic PC1/3 expression and is driven by a lack of anti-inflammatory glucocorticoids due to an impairment in the hypothalamic-pituitary-adrenal axis.

The proprotein convertase subtilisin/kexin type 1 (*PCSK1*, PC1/3) protease is expressed in endocrine and neuronal tissues and post-translationally processes relatively inactive precursor peptides and hormones into their active mature form. Peptide precursors in metabolic tissues targeted by PC1/3 include proinsulin and proglucagon (yielding glucagon and glucagon-like peptide 1 [GLP-1]) in the pancreas and proopiomelanocortin (POMC) (yielding adrenocorticotropic hormone [ACTH]) in the pituitary [1]. Further, PC1/3 mRNA and protein expression were also detected in B cells and T cells isolated from inflamed rat lymph nodes [2], primary rat alveolar macrophages, mouse peritoneal macrophages and a rat alveolar macrophage cell line [3-5], mouse splenocytes and lymphocytes [6], in human monocyte-derived macrophages and human monocytic leukemia cell line THP-1 [7], as well as liver-infiltrating leukocytes [4]. RNA and protein of the related prohormone convertase 2 (PC2) and carboxypeptidase E (CPE) were also found in B and T cells as well as macrophages of rat and human origin [2, 7]. RNA and protein of the PC1/3 targets POMC and its downstream product beta endorphin as well as proenkephalin were isolated from B cells and T cells [4, 8-10]. Thus, the prohormone convertase machinery, as well as related peptide precursors, were shown to be expressed in immune cells.

*Pcsk1* global knockout mice show increased levels of circulating pro-inflammatory cytokines in response to lipopolysaccharide (LPS) and nonhazardous doses of LPS induce septic shock and death [5]. Since spleens are enlarged, and peritoneal macrophages isolated from *Pcsk1* global knockout mice hypersecrete pro-inflammatory cytokines, it is conceivable that PC1/3 in immune cells processes and thus activates an anti-inflammatory peptide precursor. Indeed, deletion of PC1/3 in an alveolar macrophage cell line also induced a pro-inflammatory phenotype [11].

However, PC1/3 plays a pleiotropic role in regulation of the immune system. As such, PC1/3 processes several precursors of peptides involved in the hypothalamic-pituitary-adrenal stress axis, which is a key component of the host response to sepsis via production and secretion of anti-inflammatory glucocorticoids. Several feedback loops originating from the adrenals and peripheral immune cells further regulate the hypothalamic-pituitary-adrenal axis. The complexity of the interaction between the immune compartment and steroids is reflected by conflicting data around adrenal insufficiency and treatment with steroids for severe bacterial infections [12-14].

The aim of the current study was to identify the proposed anti-inflammatory peptide expressed in immune cells that is processed by PC1/3 or to find an alternative explanation for the enhanced immunity of PC1/3 knockout mice.

## Materials and Methods

### Mice

*Pcsk1* ^fl/fl^ mice (B6N-*Pcsk1*^tm1Boe^) were made by introducing loxP sites around exon 5 of the *Pcsk1* gene as previously described [15]. Conditional PC1/3 knockout mice were made by crossing *Pcsk1*^fl/fl^ mice with the following Cre-driver mice: *Vav*-iCre [16] (B6N.Cg-*Commd10^Tg(Vav1-icre)A2Kio^*, Jax strain: 018968, RRID:IMSR_JAX:018968); *Lyz2*-Cre [17] (B6N.129P2-*Lyz2^tm1(cre)Ifo^*, Jax strain: 004781, RRID:IMSR_JAX:004781); *Pomc*-Cre [18] (B6N.FVB-Tg(Pomc-cre)1Lowl, Jax strain 010714, RRID:IMSR_JAX:010714); or *UBC*-Cre [19] (B6N.Cg-Ndor1^Tg(UBC-cre/ERT2)1Ejb^, Jax strain:007001, RRID:IMSR_JAX:007001). *UBC*-Cre *Pcsk1*^fl/fl^ and control mice received 125 mg/kg tamoxifen (MedChem Express, HY-13757A) dissolved in ethanol and corn oil on Monday, Wednesday, and Friday by oral gavage. Wild-type mice (C57BL/6N or Swiss) were obtained from our in-house breeding (originally obtained from Charles River, Sulzfeld, Germany). Global *Pcsk1* knockout mice were made by crossing *Pcsk1*^fl/fl^ mice to *Cmv*-Cre [20] (B6N.Cg-Tg(CMV-cre)1Cgn, Jax strain:006054, RRID:IMSR_JAX:006054) mice with subsequent crossing to wild-type Swiss mice for 10 generations. Mice were housed in a specific pathogen-free facility at the University of Basel in groups of 2 to 6 mice per cage in individually ventilated cages (Indulab, Gams, Switzerland). The temperature (21 ± 2 °C), humidity (50%-60%) and light cycle (lights on 06:00-18:00 h) were controlled. Food was plant-based chow (3436, Kliba Nafag, Kaiseraugst, Switzerland) and water was provided ad libitum. All studies were done using littermate controls. Whenever possible, experiments were done in a blinded fashion. All animal experiments were performed in accordance with the federal laws of Switzerland and approved by the cantonal and institutional authorities of Basel.

### Breeding and Genotyping

Pups were toe-clipped between 6 and 10 days of age. Biopsies were cooked in 50 mM NaOH at 98 °C for 1 hour, followed by addition of 50 μL 1 M Tris-HCl. Then 1.25 μL was used for the polymerase chain reaction (PCR) reaction employing GoTaq G2 DNA Polymerase (M7845, Promega, Dübendorf, Switzerland) with primers (Microsynth, Balgach, Switzerland) on a Biometra TRIO thermocycler (Bartelt GmbH, Innsbruck, Austria) and visualized on self-made agarose gels using electrophoresis. Primer: *Cre* (F: 5′-GCA CTG ATT TCG ACC AGG TT-3′, R: 5′-CCC GGC AAA ACA GGT AGT TA-3′), *Actb* (F: 5′-GAC ATG CAA GGA GTG CAA GA-3′, R: 5′-TGT TAC CAA CTG GGA CA-3′), Flx *Pcsk1* (P1: 5′-CAA CAT CAA GAT GAG CAG CAC-3′, P2: 5′-AGA CTC CTT ACC GCC TAC TG-3′, P3: 5′-AAA CTT TCC CTG TCC ATC CTC-3′), i*Cre* (F: 5′-AGA TGC CAG GAC ATC AGG AAC CTG-3′, R: 5′-ATC AGC CAC ACC AGA CAC AGA GATC-3′).

### Body Weight Development

Body weight was measured weekly using the dynamic weighing feature, which averages measurements taken over a 5-second period (Scout STX, Ohaus, Nänikon, Switzerland). To increase readability, not all body weight data are depicted in the figures.

### Glycemia

Blood glucose was measured with a glucometer (FreeStyle Freedom Lite, Abbott, Baar, Switzerland) from a drop of blood obtained from a small incision into the tail vein.

### ACTH Stimulation Test

Mice were handled and accustomed to the restrainer daily for at least 2 weeks to minimize stress responses. At 9:00, blood was collected by tail-tip bleeding. Then 10 μg/kg synthetic human ACTH (Synacthen, 60009003, Future Health Pharma GmbH, Wetzikon, Switzerland) was injected intraperitoneally and one hour later, another blood sample was drawn. Plasma corticosterone was assessed by an enzyme-linked immunosorbent assay (ELISA) (ADI-900-097, Enzo Life Sciences, Lausen, Switzerland, RRID:AB_2307314).

### Circulating Interleukin (IL)-1β Levels Following In Vivo LPS Stimulation

Paracetamol (3.5 mg/mL) was added to the drinking water 24 hours prior to the experiment. Mice were injected intraperitoneally with LPS from *E. coli* (O111:B4, Sigma-Aldrich, Buchs, Switzerland) diluted in saline. Doses used are indicated in the figure legends. Blood glucose and body weight was measured at time points 0 and 24 hours after LPS injection, as described above. Blood was collected in a hematocrit capillary (9100275, Hirschmann, Eberstadt, Germany) at time points 0, 4, 8, and 24 hours after LPS injection and transferred into tubes containing 5 µL 50 mM EDTA. At the last time point, mice were euthanized with CO_2_. Blood samples were centrifuged at 10 000*g* for 5 minutes at 4 °C and plasma stored at −20 °C. Plasma IL-1β levels were measured using the V-PLEX Mouse IL-1β Kit (K152QPD, Meso Scale Discovery, Rockville, USA, RRID:AB_3532192).

### Fluorescence-Activated Cell Sorting of Peritoneal Cells and Splenocytes

Mice were euthanized with CO_2_, the skin partly removed, and 10 mL fluorescence-activated cell sorting (FACS) buffer (PBS, 0.5% BSA, 5 mM EDTA) injected into the peritoneal cavity. The peritoneum was then cut, and the lavage collected in a 50-mL Falcon tube using a funnel. Cells were then filtered through a 70 μm cell strainer, centrifuged for 5 minutes at 4 °C at 650*g*, washed once, and resuspended in 50 μL FACS buffer. In case of a visible contamination with erythrocytes, cell pellets were incubated in red cell lysis buffer (154 mM NH_4_Cl, 10 mM KHCO_3_, 0.1 mM EDTA) for 2 minutes, washed in FACS buffer, and this procedure was repeated if needed. Cells were incubated for 15 minutes in the presence of CD16/CD32 Fc receptor blocker, followed by 30 minutes incubation with fluorochrome-labeled antibodies. Cells were washed 3 times in 500 μL FACS buffer, nuclei stained with 4′,6-Diamidino-2-Phenylindole, Dilactate (DAPI) just prior to cell sorting with a BD FACS Melody (DB Biosciences, Allschwil, Switzerland). Splenocytes were isolated by smashing freshly isolated spleens through a 40-μm cell strainer, followed by removal of the supernatant and incubation of the cells in 2 mL red cell lysis buffer for 5 minutes. The reaction was stopped by addition of 8 mL FACS buffer and the tube centrifuged for 5 minutes at 4 °C at 650*g*, the supernatant removed, and the pellet resuspended in 100 μL FACS buffer containing fluorochrome-labeled antibodies. After a 30 minutes incubation, cells were washed 3 times in 500 μL FACS buffer, and nuclei were stained with DAPI just prior to cell sorting with a BD FACSARIA III Cell Sorter (DB Biosciences, Allschwil, Switzerland). Flow cytometry data was analyzed by FlowJo (v10.6.1, FlowJo LLC, RRID:SCR_008520). Antibodies: Anti-mouse CD45 PE Cy7 (eBioscience, 25-0451-82, RRID:AB_2734986, 2 µg/mL), Anti-mouse F4/80 PE (eBioscience, 12-4801-82, RRID:AB_465923, 2 µg/mL), Anti-mouse CD11b PerCP-Cyanine5.5 (eBioscience, 45-0112-80, RRID:AB_953560, 2 µg/mL), Alexa Fluor® 488 anti-mouse CD19 Antibody (BioLegend, 115521, RRID:AB_389307, 5 µg/mL), Purified anti-mouse CD16/32 Recombinant (peritoneal cells) (BioLegend, 158302, RRID:AB_2876546, 20 µg/mL), Brilliant Violet 510™ anti-mouse Ly-6G Antibody (BioLegend, 127633, RRID:AB_2562937, 4 µg/mL), DAPI (BioLegend, 422801, 3 µg/mL), CD45 Alexa Fluor 700 (BioLegend, 103128, RRID:AB_493715, 8 µg/mL), CD3 Brilliant Violet 510 (BioLegend, 100234, RRID:AB_2562555, 10 µg/mL), Purified Rat Anti-Mouse CD16/CD32 (FcBlock) (splenocytes) (BD Biosciences, 553142, RRID:AB_394657, 10 µg/mL).

### Glucocorticoid Receptor Blockade In Vivo

Wild-type mice (C57BL6/N) were orally gavaged with 500 mg/kg mifepristone (M8046, Sigma-Aldrich, Buchs, Switzerland) dissolved in ethanol/corn oil or with the corresponding vehicle (ethanol/corn oil). One hour later, LPS was injected. Mice were euthanized with CO_2_ 24 hours after LPS injection. Blood was collected from the tail tip prior to LPS injection as well as 4, 8, and 24 hours post-injection. Blood was added to tubes containing EDTA, centrifuged for 10 000*g* for 5 minutes at 4 °C, and the plasma was stored at −20 °C. IL-1β was assessed as described above.

### Steroid Pretreatment

Mice were injected intraperitoneally with 5 mg/kg dexamethasone (D1756, Sigma-Aldrich, Buchs, Switzerland), dissolved in methanol/saline or with the corresponding vehicle (methanol/saline), followed by intraperitoneal injection of LPS 30 minutes later. Mice were euthanized with CO_2_ 24 hours after LPS injection. Blood (25 μL) was collected from the tail tip prior to LPS injection as well as 4, 8, and 24 hours post-injection. Blood was added to tubes containing 2.5 μL 50 mM EDTA, centrifuged for 10 000*g* for 5 minutes at 4 °C, and the plasma was stored at −20 °C. IL-1β was assessed as described above.

### RNA Isolation, Reverse Transcription, and Quantitative PCR

Cells were lysed in 350 μL lysis buffer, frozen at −80 °C, and RNA was isolated according to the manufacturer's protocol (NucleoSpin, 740955, Macherey Nagel, Oensingen, Switzerland). RNA was reverse transcribed using random hexamers and GoScript reverse transcriptase (A278A, Promega, Dübendorf, Switzerland) according to the manufacturer's instructions. TaqMan quantitative real-time PCR was performed using GoTaq Probe kits (A610A, Promega, Dübendorf, Switzerland) and TaqMan probes (Thermo Fisher Scientific, Waltham, USA). The samples were amplified and analyzed in a ViiA7 Real-Time PCR System (Thermo Fisher Scientific). Gene expression was calculated by the comparative 2^−ΔΔ^ cycle threshold method using *Actb* as a housekeeping gene. Taqman Assays used: *Actb* (Mm00607939_s1), *Pcsk1* (Mm01345254_m1), *Pcsk2* (Mm00500974_m1), *Il1b* (Mm00434228_m1), *PCSK1* (Hs01026107_m1), *PCSK2* (Hs00159922_m1), and *IL1B* (Hs00174097_m1).

### Human Peripheral Blood Monocytes

Human peripheral blood monocytes (PBMCs) were isolated from human whole blood by density gradient centrifugation using BD Vacutainer CPT tubes. These samples were the baseline (drug-naïve) samples of an unrelated study at the University Hospital Basel study registered at ClinicalTrials.gov (NCT05167084). Study participants consented to donating blood for explorative research. The study protocol was approved by The Ethics Commission of Northwestern and Central Switzerland (EKNZ). PBMCs were plated at a density of 100 000 cells per well in a 96-well-plate in RPMI medium containing 10% FBS and standard antibiotics. 24 hours later and every 48 hours thereafter, medium was replaced with medium containing LPS. RNA was isolated after the indicated culture time as described above.

### Further Software

The manuscript was prepared using Microsoft Office version 16.

### Statistical Analyses

Data are presented as mean ± SEM. Two groups were compared using the Mann-Whitney *U* test, 3 or more groups by one-way ANOVA with a Holm-Sidak post hoc test and data comparing several timepoints by two-way ANOVA with a Holm-Sidak post hoc test. Statistical analyses were conducted with Prism v9.2 (GraphPad Software, San Diego, CA, USA, RRID:SCR_002798).

## Results

### *Pcsk1* Global Knockout Mice but Not Immune Cell-Specific *Pcsk1* Knockout Mice Show a Pro-Inflammatory Phenotype

**Figure 1.**
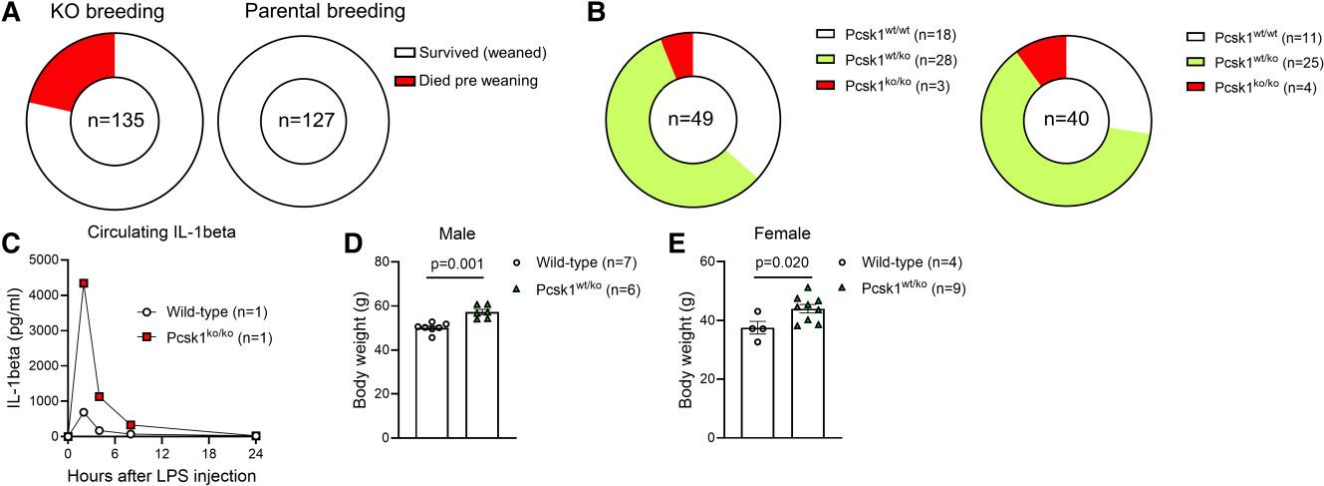
High mortality and hyperinflammation in whole-body PC1/3 knockout mice. (A) Number of mice that reached or died prior to weaning (21 days of age) obtained from intercrossing *Pcsk1*^wt/ko^ mice (left) or breeding *Pcsk1*^wt/ko^ to wild-type mice (right) on a Swiss genetic background. (B) Genotype distribution in male (left) and female (right) pups that died between 0 and 21 days of age obtained from intercrossing *Pcsk1*^wt/ko^ mice. (C) Circulating IL-1β levels before and 2, 4, 8, and 24 hours after injection of 40 μg/kg LPS in a 12-week-old female *Pcsk1* global knockout and a wild-type littermate mouse. (D-E) Body weight of male (D) and female (E) 24-week-old *Pcsk1*^wt/ko^ and *Pcsk1*^wt/wt^ littermate mice.

*Pcsk1* global knockout mice are runted and show a high mortality rate due to developmental defects [21]. Further, in response to a nonhazardous dose of LPS, these mice die due to an uncontrolled septic shock [5]. We have previously produced PC1/3 knockout mice but found these mice to not be viable on a C57BL/6N genetic background [22]. Therefore, we now crossed mice carrying loxP sites in the *Pcsk1* allele [15] with mice expressing Cre recombinase under the control of the *cmv* promoter and backcrossed the resulting deletion allele onto an outbred Swiss genetic background. We then intercrossed heterozygous mice to produce knockout and wild-type littermate offspring. In this colony, 21% of pups did not reach weaning age (21 days), while in the parental line (crossing *Pcsk1*^wt/ko^ to wild-type Swiss females) all pups survived (Fig. 1A). Of the pups that died pre-weaning, 28% were knockouts and 66% died too early to be biopsied. Of the mice that reached weaning age, 6% of the females and 10% of males were knockouts (expected: 25%) (Fig. 1B). However, most of these knockout mice died shortly after weaning. These data suggest that PC1/3 knockout mice die within the first days of life. In fact, only one female knockout mouse reached adult age. To confirm the previously published hyperinflammatory phenotype of *Pcsk1* null mice [5], we injected a low dose of LPS and found the knockout mouse to have exaggerated levels of circulating IL-1β in response to this challenge (Fig. 1C). Unlike in homozygous knockouts, viability in heterozygous littermates was normal and these mice showed increased body weights compared to wild-type littermate controls, as in published findings for the original *Pcsk1* null mouse [21] (Fig. 1D and 1E). Thus, our PC1/3 knockout mouse reproduces the immunologic and metabolic phenotype of the previously published *Pcsk1* null mouse [5].

**Figure 2.**
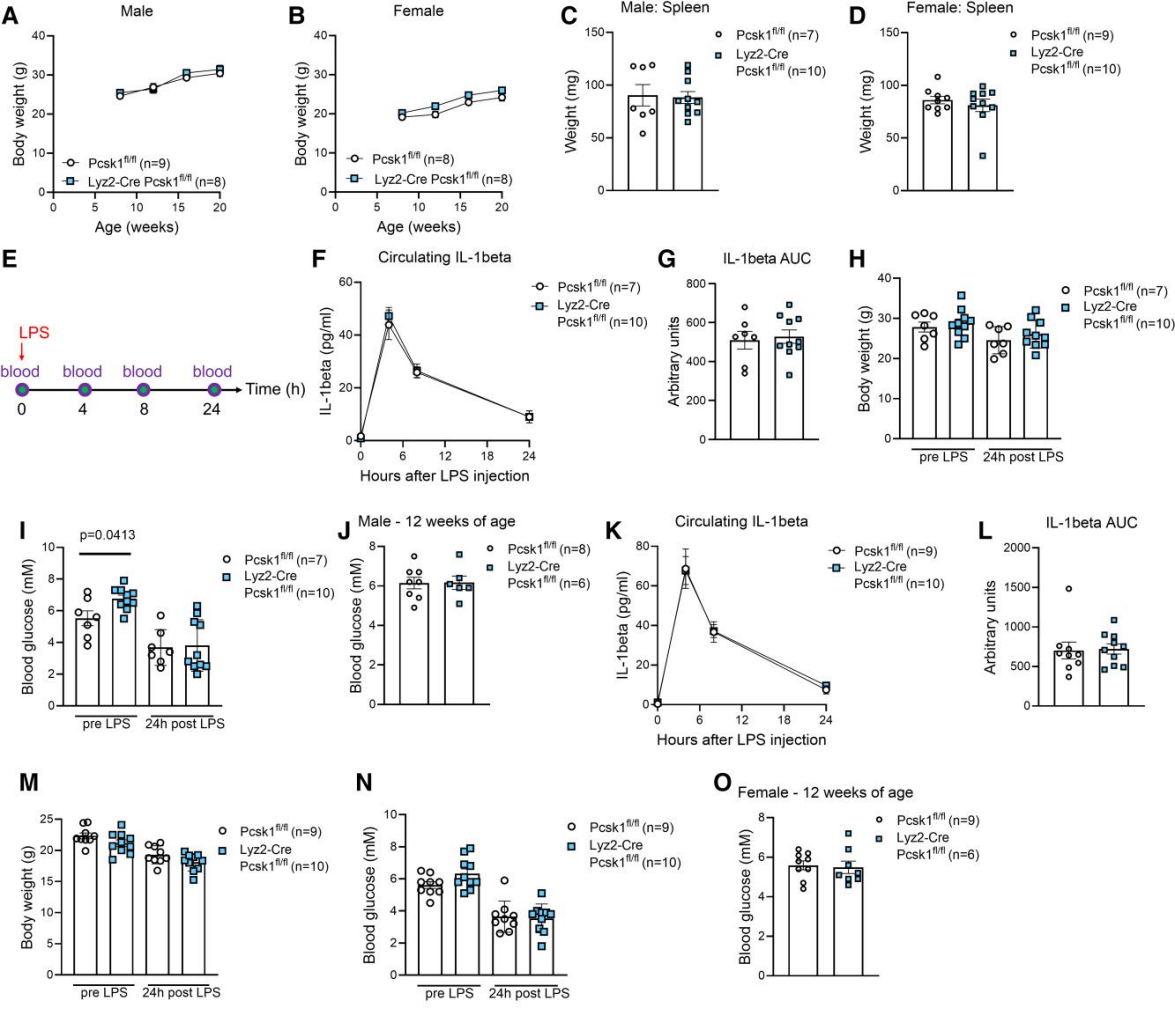
Myeloid cell-specific PC1/3 knockout mice are not hyperinflammatory. A-B, Body weight development in male (A) and female (B) *Lyz2*-Cre *Pcsk1*^fl/fl^ mice. C-D, Spleen weight of 12-week-old male (C) and female (D) *Lyz2*-Cre *Pcsk1*^fl/fl^ mice. E, Schematic representation of endotoxemia experiment. F-G, Circulating levels of IL-1β (F) and respective AUC (G) in 12-week-old male *Lyz2*-Cre *Pcsk1*^fl/fl^ mice injected with 2 mg/kg body weight LPS at timepoint 0. H-I, Body weight (H) and glycemia (I) before and 24 hours after injection of 2 mg/kg body weight LPS in 12-week-old male *Lyz2*-Cre *Pcsk1*^fl/fl^ mice. (J) Blood glucose in 12-week-old male *Lyz2*-Cre *Pcsk1*^fl/fl^ mice obtained from an additional cohort of untreated mice. K-L, Circulating levels of IL-1β (K) and respective AUC (L) in 12-week-old female *Lyz2*-Cre *Pcsk1*^fl/fl^ mice injected with 2 mg/kg body weight LPS at timepoint 0. M-O, Body weight (M) and glycemia (N) before and 24 hours after injection of 2 mg/kg body weight LPS as well as in an additional untreated cohort of mice (O) in 12-week-old female *Lyz2*-Cre *Pcsk1*^fl/fl^ mice. Abbreviations: AUC, area under the curve; LPS, lipopolysaccharide.

Since immune cells isolated from *Pcsk1* global knockout mice and genetic knockout of *Pcsk1* in an immortalized immune cell line showed a pro-inflammatory phenotype [5, 23], we reasoned that PC1/3 might process and thus activate an anti-inflammatory peptide in immune cells. We therefore bred constitutive myeloid-specific *Pcsk1* knockout mice (*Lyz2*-Cre *Pcsk1*^fl/fl^). Mice of both sexes had body weights comparable to their controls (Fig. 2A and 2B). In contrast to *Pcsk1* global knockout mice, myeloid-specific *Pcsk1* knockout mice had unaltered spleen sizes compared to their littermate controls (Fig. 2C and 2D). We next challenged these mice with a low dose of LPS (Fig. 2E) and found that circulating IL-1β concentrations and body weight development were unchanged in male myeloid-specific *Pcsk1* knockout mice compared to controls (Fig. 2F-2H). Baseline glycemia in males was increased (Fig. 2I). To test whether this observation was robust, we analyzed an additional cohort of mice that previously did not undergo any in vivo intervention and found that they did not present hyperglycemia (Fig. 2J), suggesting that this finding is not reproducible. Similarly, female myeloid-specific *Pcsk1* knockout mice showed unaltered circulating IL-1β levels in response to low-dose LPS (Fig. 2K and 2L) and body weight was also unchanged between genotypes (Fig. 2M). Glycemia before and after injection of LPS as well as in an additional unhandled cohort of mice was comparable between genotypes (Fig. 2N and 2O).

**Figure 3.**
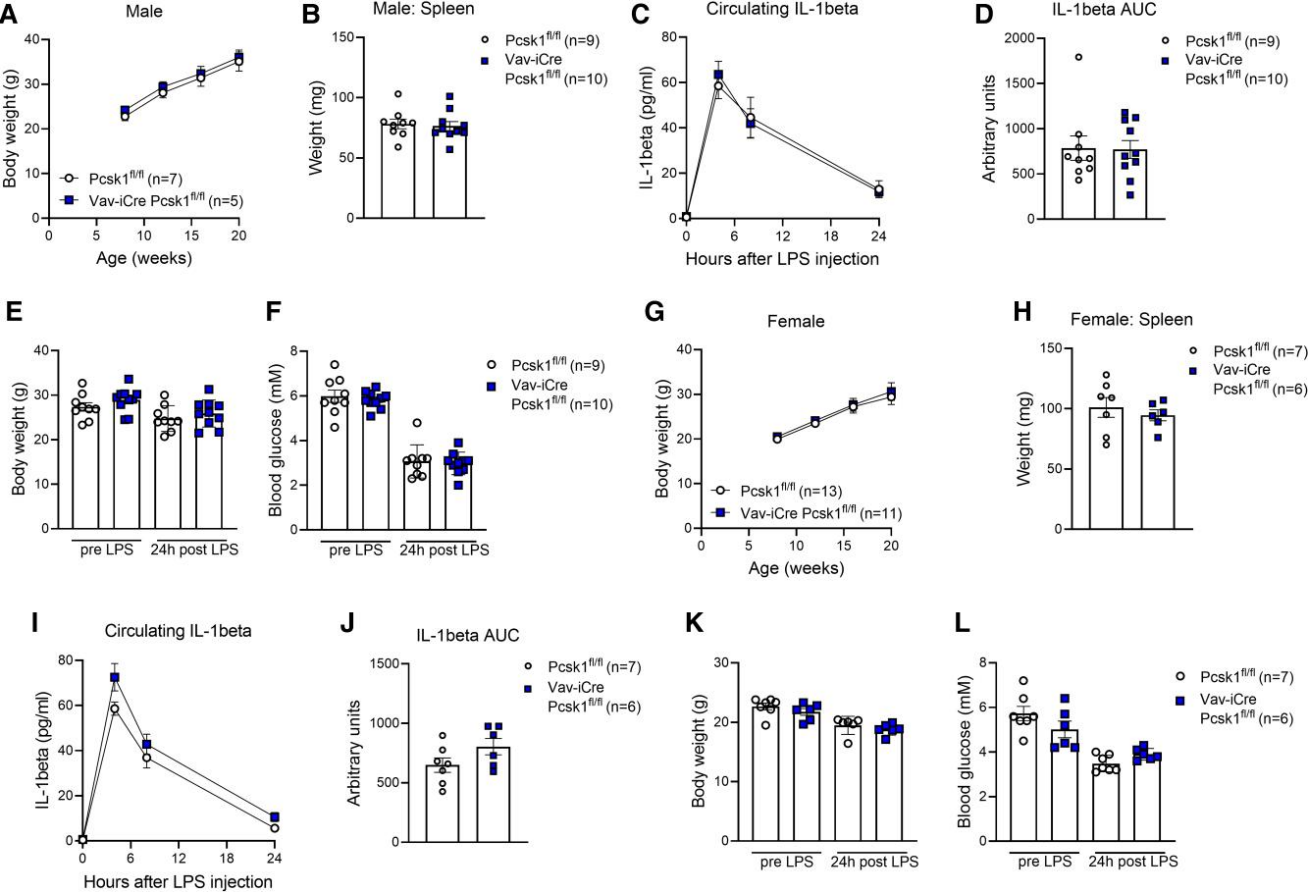
Hematopoietic cell-specific PC1/3 knockout mice are not hyperinflammatory. (A) Body weight development in male *Vav*-iCre *Pcsk1*^fl/fl^ mice. (B) Spleen weight of 12-week-old male *Vav*-iCre *Pcsk1*^fl/fl^ mice. C-D, Circulating levels of IL-1β (C) and respective AUC (D) in 12-week-old male *Vav*-iCre *Pcsk1*^fl/fl^ mice injected with 2 mg/kg body weight LPS at timepoint 0. E-F, Body weight (E) and glycemia (F) before and 24 hours after injection of 2 mg/kg body weight LPS in 12-week-old male *Vav*-iCre *Pcsk1*^fl/fl^ mice. (G) Body weight development in female *Vav*-iCre *Pcsk1*^fl/fl^ mice. (H) Spleen weight of 12-week-old female *Vav*-iCre *Pcsk1*^fl/fl^ mice. I-J, Circulating levels of IL-1β (I) and respective AUC (J) in 12-week-old female *Vav*-iCre *Pcsk1*^fl/fl^ mice injected with 2 mg/kg body weight LPS at timepoint 0. K-L, Body weight (K) and glycemia (L) before and 24 hours after injection of 2 mg/kg body weight LPS in 12-week-old female *Vav*-iCre *Pcsk1*^fl/fl^ mice. Abbreviation: AUC, area under the curve; LPS, lipopolysaccharide.

These results were rather puzzling, considering the fact that deletion of PC1/3 in an alveolar macrophage cell line induced a pro-inflammatory phenotype [3, 11]. Therefore, we decided to expand the targeted cells to all hematopoietic tissues and intercrossed *Pcsk1* floxed mice with mice expressing iCre recombinase under the control of the mouse HS21/45 control region. Male *Vav*-iCre *Pcsk1*^fl/fl^ mice had a similar body weight development and spleen weight compared to their controls (Fig. 3A and 3B). Circulating IL-1β levels in response to LPS, as well as body weight and glycemia before and after LPS challenge, were also indistinguishable between genotypes (Fig. 3C-3F). Similarly, female *Vav*-iCre *Pcsk1*^fl/fl^ mice also did not differ from their controls in terms of body weight development, spleen weight, LPS-induced circulating IL-1β as well as body weight and glycemia before and after LPS challenge (Fig. 3G-3L). These data show that lack of PC1/3 in immune cells does not contribute to the pro-inflammatory phenotype observed in *Pcsk1* global knockout mice.

### *Pomc*-Specific *Pcsk1* Ablation Reproduces Pro-Inflammatory Phenotype of *Pcsk1* Global Knockout Mice

**Figure 4.**
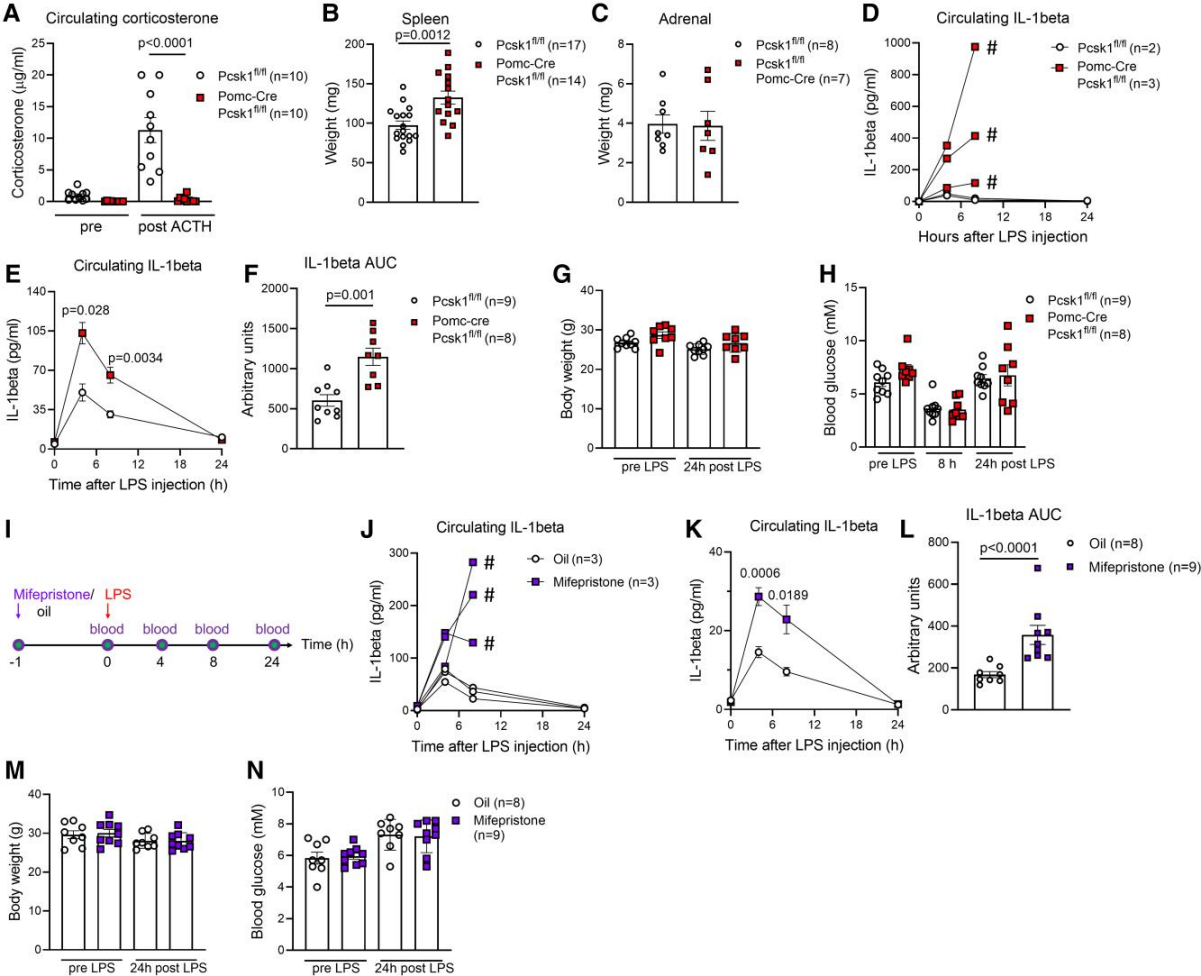
*Pomc*-specific *Pcsk1* ablation or blocking glucocorticoid receptors reproduces the pro-inflammatory phenotype of *Pcsk1* null mice. (A) Circulating corticosterone levels before and after injection of 10 μg/kg body weight ACTH in 26- to 30-week-old male *Pomc*-Cre *Pcsk1*^fl/fl^ mice. B-C, Spleen (B) and adrenal (C) weight of 12- to 14-week-old male *Pomc*-Cre *Pcsk1*^fl/fl^ mice. (D) Circulating levels of IL-1β before and after injection of 2 mg/kg body weight LPS in 12-week-old male *Pomc*-Cre *Pcsk1*^fl/fl^ mice. # indicates that a mouse reached a humane endpoint. E-F, Circulating IL-1β (E) and respective AUC (F) before and after injection of 40 μg/kg body weight LPS in 12-week-old male *Pomc*-Cre *Pcsk1*^fl/fl^ mice. G-H, Body weight (G) and glycemia (H) before and 24 hours after injection of 40 μg/kg body weight LPS in 12-week-old male *Pomc*-Cre *Pcsk1*^fl/fl^ mice. I, Schematic overview of mifepristone experiment. J, Circulating levels of IL-1β before and after injection of 2 mg/kg body weight LPS in 12-week-old male wild-type mice preinjected with 500 mg/kg mifepristone or oil. # indicates that a mouse reached a humane endpoint. K-L, Circulating IL-1β (K) and respective AUC (L) before and after injection of 40 μg/kg body weight LPS in 14- to 15-week-old male wild-type mice preinjected with 500 mg/kg mifepristone or oil. M-N, Body weight (M) and glycemia (N) before and 24 hours after injection of 40 μg/kg body weight LPS in 14- to 15-week-old male wild-type mice preinjected with 500 mg/kg mifepristone or oil. Abbreviations: AUC, area under the curve; LPS, lipopolysaccharide.

Since the detrimental pro-inflammatory phenotype of *Pcsk1* global knockout mice was not reproducible in immune cell-specific *Pcsk1* knockout mice, we reasoned that there must be another anti-inflammatory PC1/3-cleaved peptide that modulates sepsis susceptibility. The hypothalamic-pituitary-adrenal stress axis is well recognized for its initiation of anti-inflammatory action by regulating the secretion of steroids from the adrenals. Several components of this signaling cascade are dependent on PC1/3, including processing of POMC to ACTH. Therefore, we next deleted PC1/3 from POMC-expressing tissues (*Pomc*-Cre *Pcsk1*^fl/fl^). We previously showed that female *Pomc*-Cre *Pcsk1*^fl/fl^ mice suffer from adrenal insufficiency [22]. Similarly, male mice were unable to mount an appropriate steroid response when injected with ACTH (Fig. 4A), suggesting that the hypothalamic-pituitary-adrenal axis is impaired in these mice. Spleen weight was increased while adrenal weight was unchanged (Fig. 4B and 4C). Injection of a low dose of LPS triggered a rapid rise of circulating IL-1β in *Pomc*-Cre *Pcsk1*^fl/fl^ mice but not in control mice (Fig. 4D). These animals were euthanized at the 8-hour timepoint due to reaching a humane endpoint. Next, we injected 50-fold less LPS and found that *Pomc*-Cre *Pcsk1*^fl/fl^ mice survived, but still had increased levels of circulating IL-1β in response to LPS (Fig. 4E and 4F). This low dose of LPS did not induce changes in body weight or glycemia between genotypes (Fig. 4G and 4H).

### Blocking Glucocorticoid Receptors Mimics the Phenotype of *Pomc*-Cre *Pcsk1*^fl/fl^ and *Pcsk1* Global Knockout Mice

To pinpoint these finding to the pituitary-adrenal axis, we next interfered downstream of the POMC processing pathway, at the level of steroid signaling (Fig. 4I). Injecting LPS after an acute blockade of glucocorticoid receptors with mifepristone in wild-type mice induced IL-1β hypersecretion (Fig. 4J). Similarly to the experiment described above, mice were euthanized at the 8-hour timepoint due to reaching a humane endpoint. Reducing the dose of LPS by 50-fold enabled all mice to survive but circulating IL-1β levels were still increased in mice pretreated with mifepristone compared to controls (Fig. 4K and 4L). Body weight and glycemia did not differ between genotypes during the experiment (Fig. 4M and 4N). Taken together, these data show that mice with impaired POMC processing or blocked glucocorticoid receptors phenocopy the immunological phenotype of whole-body PC1/3 knockout mice.

### Steroid Therapy Prevents LPS-Induced Sepsis in *Pomc*-Cre *Pcsk1*^fl/fl^ Mice

**Figure 5.**
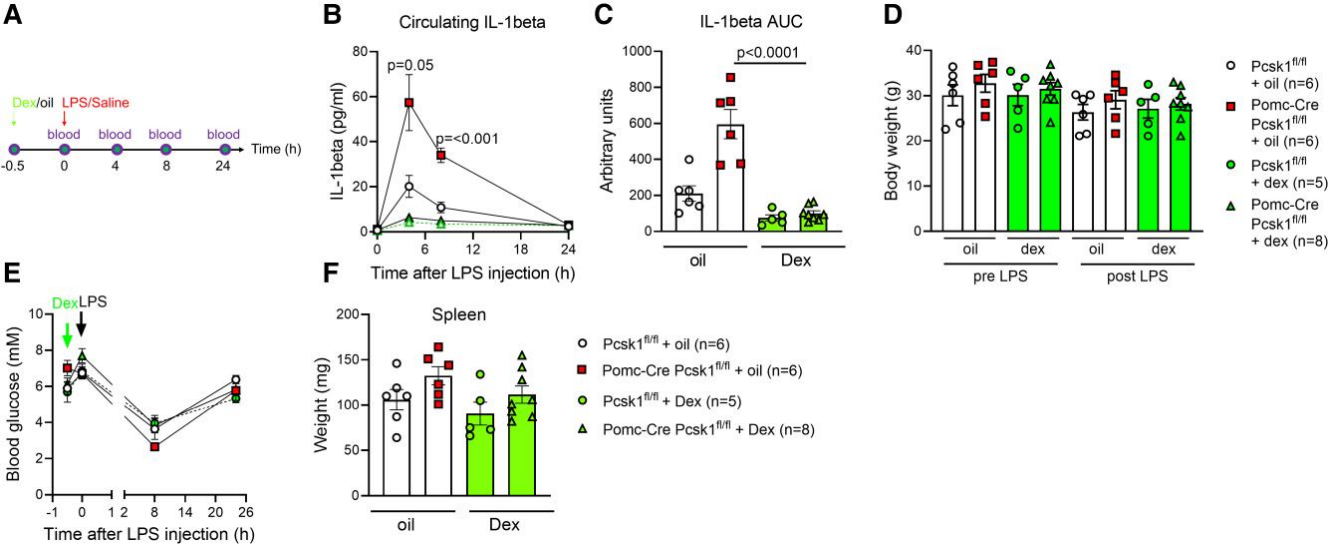
Steroid therapy prevents LPS-induced sepsis in *Pomc*-Cre *Pcsk1*^fl/fl^ mice. (A), Schematic presentation of dexamethasone experiment. B-C, Circulating IL-1β levels (B) and respective AUC (C) before and after injection of 80 μg/kg body weight LPS in 12-week-old male *Pomc*-Cre *Pcsk1*^fl/fl^ mice preinjected with 5 mg/kg dexamethasone or oil. D-F, Body weight (D), glycemia (E) and spleen weight (F) before and after injection of 80 μg/kg body weight LPS in 12-week-old male *Pomc*-Cre *Pcsk1*^fl/fl^ mice preinjected with 5 mg/kg dexamethasone or oil. Abbreviations: AUC, area under the curve; Dex, dexamethasone; LPS, lipopolysaccharide.

Since an impaired hypothalamic-pituitary-adrenal stress axis induced exaggerated circulating IL-1β levels in response to low-dose LPS, we next investigated whether this phenotype can be prevented by reintroducing glucocorticoids. For this, *Pomc*-Cre *Pcsk1*^fl/fl^ mice were pretreated with the synthetic steroid dexamethasone and this completely prevented LPS-induced hypersecretion of IL-1β (Fig. 5A-5C). Body weight and glycemia did not differ between groups during the experiment (Fig. 5D and 5E). Spleens from *Pomc*-Cre *Pcsk1*^fl/fl^ mice tended to be heavier and dexamethasone seemed to reduce spleen weights, although this did not reach statistical significance (Fig. 5F). These data show that normalizing reduced steroid levels in mice with an impaired hypothalamic-pituitary-adrenal axis prevents LPS hypersensitivity.

### Lack of Robust PC1/3 Expression in Mouse and Human Myeloid and Hematopoietic Cells

**Figure 6.**
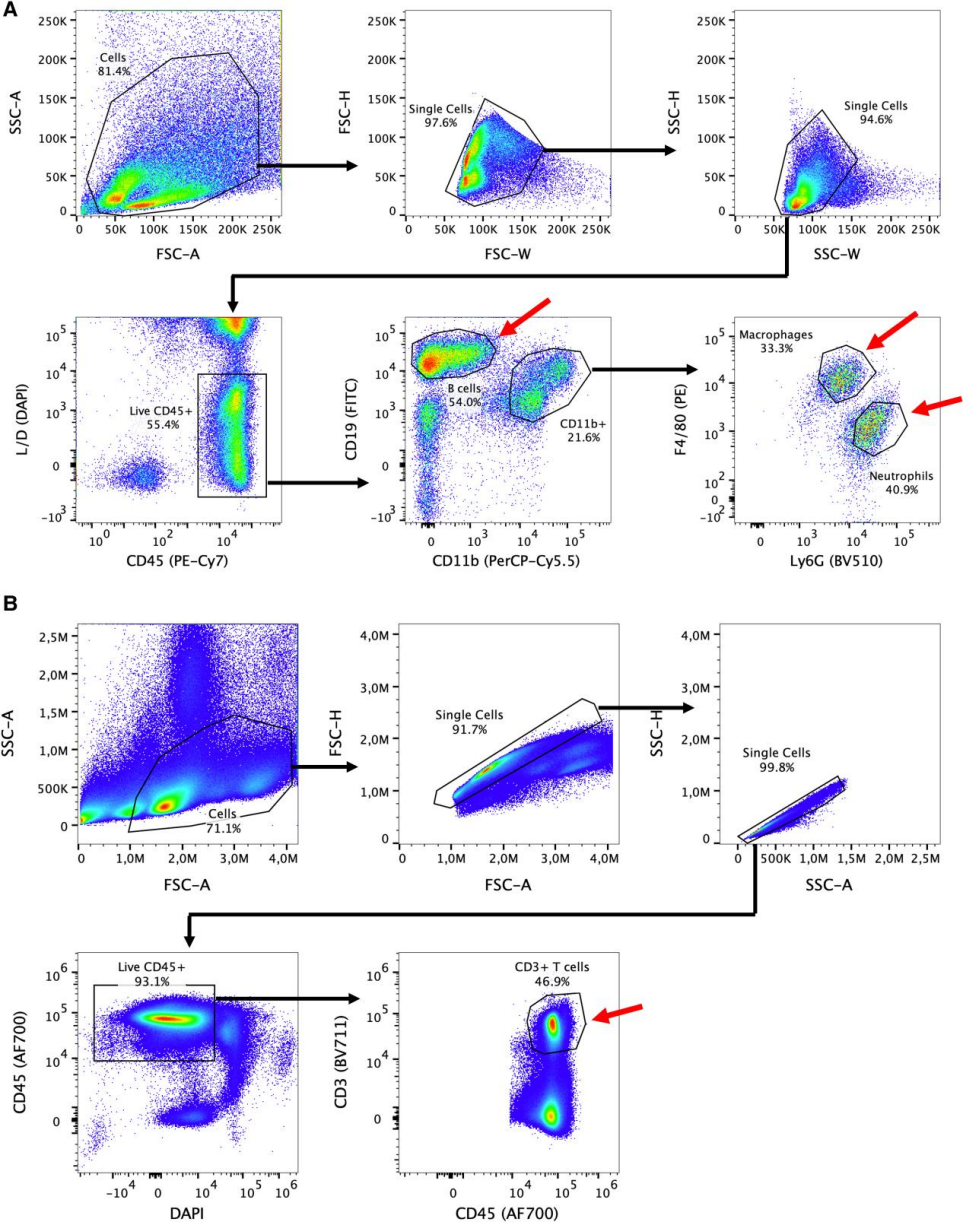
Gating strategy of peritoneal and splenic immune cells. (A) Sorting of macrophages (CD45 + CD11b^high^F4/80+), neutrophils (CD45 + CD11b^high^Ly6G+), and B cells (CD45 + CD11b^low^) from peritoneal lavage. (B) Sorting of T cells (CD45 + CD3+) from splenocytes. Arrows pointing to specific populations indicate cells that were collected for downstream analysis.

The unexpected absence of a phenotype in immune cell-specific PC1/3 knockout mice promoted us to investigate the expression pattern of PC1/3 in hematopoietic cells in more detail. First, in accordance with what was published by Refaie et al [5], wild-type C57BL/6N mice were injected with the immune-booster thioglycolate or saline, followed by injection of 2 mg/kg body weight LPS 48 hours later. Twenty-four hours later, peritoneal cells and splenocytes were isolated and macrophages (CD45 + CD11b^high^F4/80+), neutrophils (CD45 + CD11b^high^Ly6G+), B cells (CD45 + CD11b^low^), and T cells (CD45 + CD3+) collected by FACS (Fig. 6A and 6B). From these cells, RNA was isolated and analyzed by TaqMan chemistry (n = 2 independent isolations, 4 individual mice per isolation and treatment group). *Actb* was readily detectable (cycle threshold (ct) 16-18) but *Pcsk1* and *Pcsk2* were undetectable (no signal above background, 40 cycles). As a positive control, we measured *Il1b* as well as *Pcsk1* in islet lysate samples and found the ct to be 23-25 and 25-26, respectively, confirming that the assay worked. Next, we investigated immune cells from inducible whole-body PC1/3 knockout mice [22] (*UBC*-Cre^ERT2^  *Pcsk1*^fl/fl^). *Pcsk1* knockout was induced by application of tamoxifen at 8 weeks of age. Four weeks later, tissues were isolated and processed for analysis. Hypothalamic *Pcsk1* expression was reduced by 91% (ct *Actb* 20-21, ct *Pcsk1* 24-25) showing that *Pcsk1* expression was successfully ablated. However, *Pcsk1* and *Pcsk2* expression in FACS-purified macrophages, neutrophils, B cells and T cells were below the detection limit (no signal above background, 40 cycles) while the positive controls *Il1b* and *Pcsk1* in islet lysates were detectable at similar ct values as described above (for immune cells: n = 3 independent isolations, 3 to 4 individual mice per isolation/treatment group/genotype). Since alveolar macrophages are in a preactivated state and *Pcsk1* RNA and protein were found in an alveolar macrophage cell line [3], we next assessed bronchoalveolar lavage isolated from *UBC*-Cre^ERT2^  *Pcsk1*^fl/fl^ and control mice 24 hours after LPS injection (5 samples per genotype, pools of several mice) 4 weeks after knockout induction. As before, no robust signal for *Pcsk1* and *Pcsk2* was detected. The *Pcsk1* global knockout mouse used by Refaie et al [5] was on a CD1 genetic background while we isolated immune cells from wild-type and knockout mice on a C57BL/6N genetic background. To prove that our conflicting findings are not based on differences in the genetic background, we next isolated and analyzed peritoneal cells 24 hours after LPS injection from Swiss mice (the progenitor line of CD1). Again, *Pcsk1* and *Pcsk2* expression was below the detection limit while positive controls *Il1b* and *Pcsk1* from islet lysates were readily detectable (n = 3 saline, n = 3 LPS).

Several research groups reported detection of *PCSK1* in various human immune cells, at least when induced by LPS. We therefore next cultured freshly isolated human PBMCs in the presence of 0, 100, or 200 ng/mL LPS for 4 hours (n = 6 donors) and in a second series in the presence of 100 or 1000 ng/mL LPS for 72 and 144 hours (n = 3 donors). *ACTB* (ct = 22-24) and *IL1B* (ct = 18-20) were highly expressed in these samples but *PCSK1* and *PCSK2* were not detectable (no signal above background, 40 cycles). Noteworthy, robust detection of *PCSK1* and *PCSK2* from an islet lysate sample (ct 26 and 27, respectively) confirmed the validity of the assay. These data suggest that, at least under the conditions tested, immune cells do not robustly express PC1/3.

Overall, our study suggests that there is a limited role for PC1/3 in immune cells and it might actually not be expressed at all. The observed hyperinflammatory phenotype in *Pcsk1* global knockout mice is uncoupled from immune cell intrinsic PC1/3 expression and is driven by a lack of anti-inflammatory glucocorticoids based on reduced action of the hypothalamic-pituitary-adrenal axis.

## Discussion

Sepsis is a life-threatening organ dysfunction caused by a dysregulated host immune response to infection [24]. During an endotoxemia challenge, which is often used as an experimental model of sepsis, mice with global deficiency of the protease PC1/3 disproportionally increase their secretion of pro-inflammatory cytokines and die due to septic shock even when exposed to nonlethal doses of LPS [5]. PC1/3 processes and activates dozens of neuropeptides and endocrine peptide precursors making it a challenge to pinpoint precisely which pathways are involved in this dysregulated immune response to LPS in PC1/3 knockout mice. Further, studies with isolated macrophages of PC1/3 knockout mice and immune cell lines lacking PC1/3 expression both demonstrated pro-inflammatory phenotypes, thereby strongly implicating immune cells as the potential mediators of the deleterious effects of PC1/3 ablation [3, 5, 25]. We aimed to identify a potential immune cell-expressed anti-inflammatory peptide that depends on PC1/3 processing, resulting in its active form being absent in PC1/3 knockout models. However, our findings not only suggest that immune cell PC1/3 expression is not involved in sepsis pathology, but rather the lack of anti-inflammatory action of glucocorticoids stemming from adrenal insufficiency mediates the hyperinflammatory phenotype seen in PC1/3 deficient mice.

To study a potential role for PC1/3 in immune cells, we produced 2 tissue-specific PC1/3 knockout mouse lines targeting the myeloid and hematopoietic compartment, respectively. These mice did not show a metabolic nor inflammatory phenotype. When trying to quantify knockout efficiency, we were unable to robustly detect *Pcsk1* in immune cells, which we initially attributed to the reported inducible nature of PC1/3 expression in immune cells [7]. However, even when we employed published protocols to stimulate PC1/3 expression in vivo and in vitro, we failed to detect *Pcsk1* and *Pcsk2* expression in sorted macrophages, B cells, neutrophils, and T cells from various mouse models. Further, in contrast to previous reports [7], using freshly isolated human PBMCs treated in vitro with LPS, we still could not demonstrate any *PCSK1* expression in immune cells. We do not have a conclusive explanation for this apparent difference of our observations relative to the published literature. One difference between our study and all published studies mentioned in this manuscript is that we used the highly sensitive and selective TaqMan system, instead of methods which label any double-stranded DNA. Compared to the TaqMan system, fluorescent dyes such as Thiazole Green have the disadvantage that they may generate false-positive signals, especially when the expression of the target gene is very low. However, inclusion of proper negative and positive controls should preclude these issues. It is possible that prohormone convertases are induced in immune cells under certain experimental conditions that we did not meet in our studies. We designed our studies as similar as possible to the previously published reports of PC1/3 expression, but they were naturally not identical. Nevertheless, based on the absence of an LPS-induced pro-inflammatory phenotype in 2 immune cell-specific PC1/3 knockout mouse models, our data negate a biologically significant role of PC1/3 in the hematopoietic compartment in the pathogenesis of endotoxemia and sepsis.

One explanation for why transcripts are detected in immune cells which they themselves might not have expressed could be the due to the high sensitivity that modern detection methods possess. Brykczynska and colleagues recently published RNA sequencing data of various tissue resident macrophages in response to nutritional challenges [26]. Interestingly, *Pcsk1* and *Pcsk2* transcripts were found in islet macrophages but not in microglia or peritoneal macrophages. However, typical endocrine transcripts like *Ins1*, *Ins2*, *Iapp*, *Gcg*, and *Sst* were also detected, suggesting that these transcripts are traceable in immune cells because they have been phagocytosed. Further, Ying et al detected the transcripts mentioned above in macrophages isolated from both within and peripheral to pancreatic islets from high-fat diet–fed mice, supporting the notion that these signals stem from internalized endocrine cells or their products [27].

Despite the negative results obtained from immune cell-specific PC1/3 knockout mice, there must be a PC1/3 target that is implicated in the strong pro-inflammatory phenotype in whole-body PC1/3 knockout mice, as observed by Refaie et al [5] and replicated in our *Pcsk1 null* mouse model. A second potential point of contact between PC1/3 and sepsis is the hypothalamic-pituitary-adrenal axis. Several peptides that act on different levels of this stress response are processed by PC1/3, including corticotropin-releasing hormone, that is secreted from the paraventricular nucleus of the hypothalamus, which in turn stimulates the production and secretion of ACTH from the pituitary. ACTH itself is derived from its precursor POMC which depends on PC1/3 processing as well. During infection, cytokines from activated monocytes signal through the hypothalamic-pituitary axis to counteract infection by releasing anti-inflammatory steroids, such as glucocorticoids, from the adrenals. Deletion of the glucocorticoid receptor in macrophages increased the susceptibility to LPS-induced mortality [28], suggesting that the anti-inflammatory properties of glucocorticoids are at least partly mediated by directly acting on immune cells. Our data suggest that PC1/3 in immune cells is not involved in LPS-induces sepsis. However, PC1/3 deficiency could contribute to increased inflammation by impairing the hypothalamic-pituitary axis, thereby reducing the level of anti-inflammatory glucocorticoids. Indeed, ablation of PC1/3 in *Pomc*-expressing tissues phenocopied *Pcsk1* global knockout mice. Pomc-specific PC1/3 knockout mice exhibited enlarged spleens, increased levels of circulating cytokines and succumbed to a nonlethal dose of LPS. In addition, interfering with this axis at a more downstream level by acutely blocking glucocorticoid receptors pharmacologically, also resulted in a similar pro-inflammatory phenotype. Increased mortality in rodents with disrupted hypothalamic-pituitary-adrenal axis either by blocking glucocorticoid action [29] or by surgical removal of the adrenals [30] has already been reported.

Consequently, exogenous supplementation of steroids prevented this phenotype in mice with POMC-specific PC1/3 ablation. The spleen size was enlarged in *Pomc*-Cre *Pcsk1*^fl/fl^ mice already prior to stimulation with LPS. Interestingly, another mouse model with reduced levels of corticosterone, the *Clock* gene mutant mouse, also shows enlarged spleens [31]. This might suggest that glucocorticoids negatively regulate spleen size.

In summary, we show that the hyperinflammatory phenotype in global PC1/3-deficient mice is uncoupled from immune cell intrinsic PC1/3 expression. Rather, impaired POMC processing in the pituitary induces adrenal insufficiency which increases susceptibility to endotoxemia due to lack of anti-inflammatory action of glucocorticoids.

## Data Availability

Original data generated and analyzed during this study are included in this published article.

## Abbreviations

ACTH: adrenocorticotropic hormone
CPE: carboxypeptidase E
ct: cycle treshold
FACS: fluorescence-activated cell sorting
IL: interleukin
LPS: lipopolysaccharide
PBMC: human peripheral blood monocyte
PC1/3: prohormone convertase 1/3
PC2: prohormone convertase 2
PCR: polymerase chain reaction
POMC: proopiomelanocortin
